# Tuberculous granulomatous inflammation of parathyroid adenoma manifested as primary hyperparathyroidism: A case report and a review of the literature

**DOI:** 10.3892/mi.2023.109

**Published:** 2023-09-05

**Authors:** Abdulwahid M. Salih, Aras J. Qaradakhy, Shko H. Hassan, Ari M. Abdullah, Hardi Mohammed Dhahir, Sanaa O. Karim, Hawar A. Sofi, Berun A. Abdalla, Muhammad Hassan Ali, Fahmi H. Kakamad

**Affiliations:** 1 Department of Scientific Affairs, Smart Health Tower, Sulaimani, Kurdistan 46000, Iraq; 2 College of Medicine, University of Sulaimani, Sulaimani, Kurdistan 46000, Iraq; 3 Department of Radiology, Shorsh Teaching Hospital, Sulaimani, Kurdistan 46000, Iraq; 4 Department of Pathology, Sulaimani Teaching Hospital, Sulaimani, Kurdistan 46000, Iraq; 5 College of Nursing, University of Sulaimani, Sulaimani, Kurdistan 46000, Iraq; 6 Kscien Organization for Scientific Research, Sulaimani, Kurdistan 46000, Iraq

**Keywords:** parathyroid adenoma, hypercalcemia, tuberculosis, extrapulmonary tuberculosis, primary hyperparathyroidism, caseating granulomatous inflammation

## Abstract

Tuberculosis of the thyroid gland is rare, and tuberculous granulomatous inflammation of the parathyroid glands is even rarer. The present study reports a rare case of primary hyperparathyroidism caused by tuberculous granulomatous inflammation. A 58-year-old female patient presented with generalized body pain persisting for 1 year. She had a history of recurrent renal stones (>20 times) and an incidental finding of multinodular goiter involving the parathyroid on neck ultrasound. A blood analysis revealed elevated levels of serum calcium (11.26 mg/dl) and parathyroid hormone (154.7 pg/ml). The patient underwent the resection of the affected left thyroid lobe under general anesthesia. A histopathological examination revealed parathyroid adenoma with caseating granulomatous inflammation involving the adenoma with focal lymphocytic thyroiditis of the left thyroid gland. Although granulomatous parathyroid disease with parathyroid adenoma causing hypercalcemia is an extremely rare event, it can occur. The treatment of choice is surgical resection.

## Introduction

Primary hyperparathyroidism (PHPT) is one of the most common causes of hypercalcemia with various etiologies among outpatients. Parathyroid adenoma followed by parathyroid hyperplasia is a frequent cause of PHPT, whereas carcinoma of the parathyroid is the main cause of hypercalcemia in hospitalized patients (1,2).

Granulomatous disorders are a type of chronic inflammation caused by autoimmune diseases, allergies, infections, toxins, and neoplastic conditions (3). Tuberculosis (TB) and sarcoidosis are common granulomatous diseases that rarely affect the parathyroid gland and their co-occurrence with a functioning parathyroid adenoma leading to hypercalcemia is extremely uncommon (4,5). Vitamin D [1,25(OH)_2_D_3_]-mediated granulomatous disorder, along with osteoclast activation within the bones, is the leading cause of high serum levels of calcium. The majority of patients with granulomatous disease-related hypercalcemia do not present with symptoms; however, in some cases, chronic hypercalcemia may occur (6). To date, at least to the best of our knowledge, only eight cases of granulomatous inflammation of the parathyroid gland caused by TB have been reported in the literature (4,7-13).

The present study reports a rare case of parathyroid adenoma with caseating granulomatous inflammation leading to hypercalcemia. The cited articles were assessed for eligibility based on Kscien's list (14).

## Case report

### Patient information

A 58-year-old female patient with a positive history of recurrent renal calculi presented to the Head and Neck clinic at Smart Health Tower (Sulaimani, Iraq) with complaints of generalized body aches and fatigue for about a year. The patient did not have any notable previous surgical history or infection with TB.

### Clinical findings

A physical examination did not reveal any notable findings, and there was no associated palpable cervical lymphadenopathy.

### Diagnostic assessment

**Figure 1:**
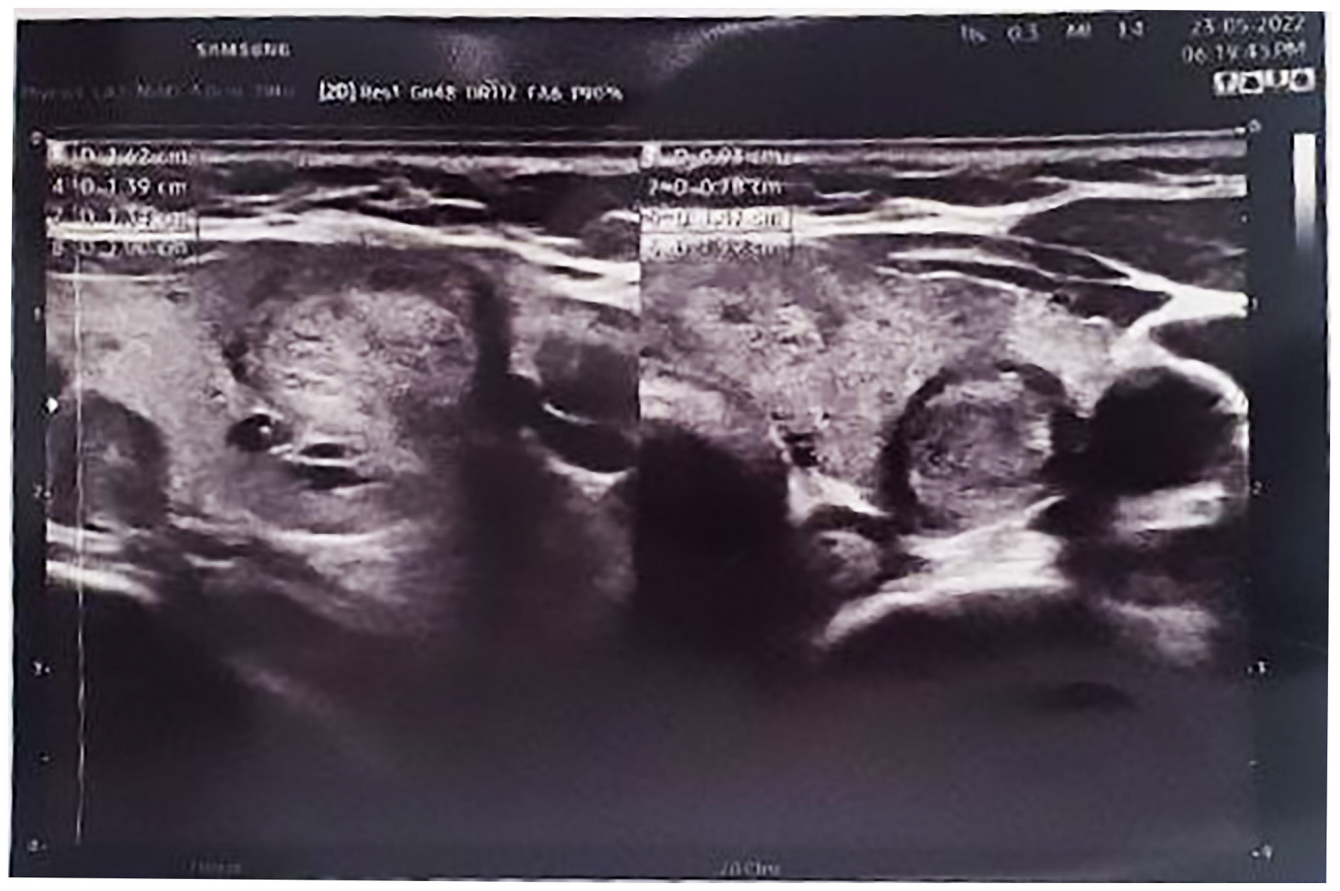
Ultrasound image illustrating a multinodular goiter with mildly suspicious, bilateral homogeneous echo texture nodules measuring 4 mm within the right thyroid gland. The left thyroid gland exhibited a non-suspicious (TR2) nodule measuring 13x9x8 mm and a mildly suspicious (TR3) nodule measuring 10x9x7 mm. Below the left lower pole of the thyroid gland, a solid hypoechoic hypovascular nodule of 20x7 mm was observed, which suggested a parathyroid adenoma. No notable cervical lymphadenopathy was observed.

The results of blood analyses revealed a high level of parathyroid hormone (PTH) (154.7 pg/ml) and serum calcium (11.26 mg/dl). An ultrasound examination of the neck revealed a multinodular goiter with mildly suspicious (TR3) bilateral homogeneous echo texture nodules measuring 4mm within the right thyroid gland. The left thyroid gland showed a non-suspicious (TR2) nodule measuring 13x9x8 mm and a mildly suspicious (TR3) nodule measuring 10x9x7 mm. Below the left lower pole of the thyroid gland, a solid hypoechoic hypovascular nodule of 20x7 mm was observed, which suggested a parathyroid adenoma. No notable cervical lymphadenopathy was observed (Fig. 1).

### Therapeutic intervention

**Figure 2:**
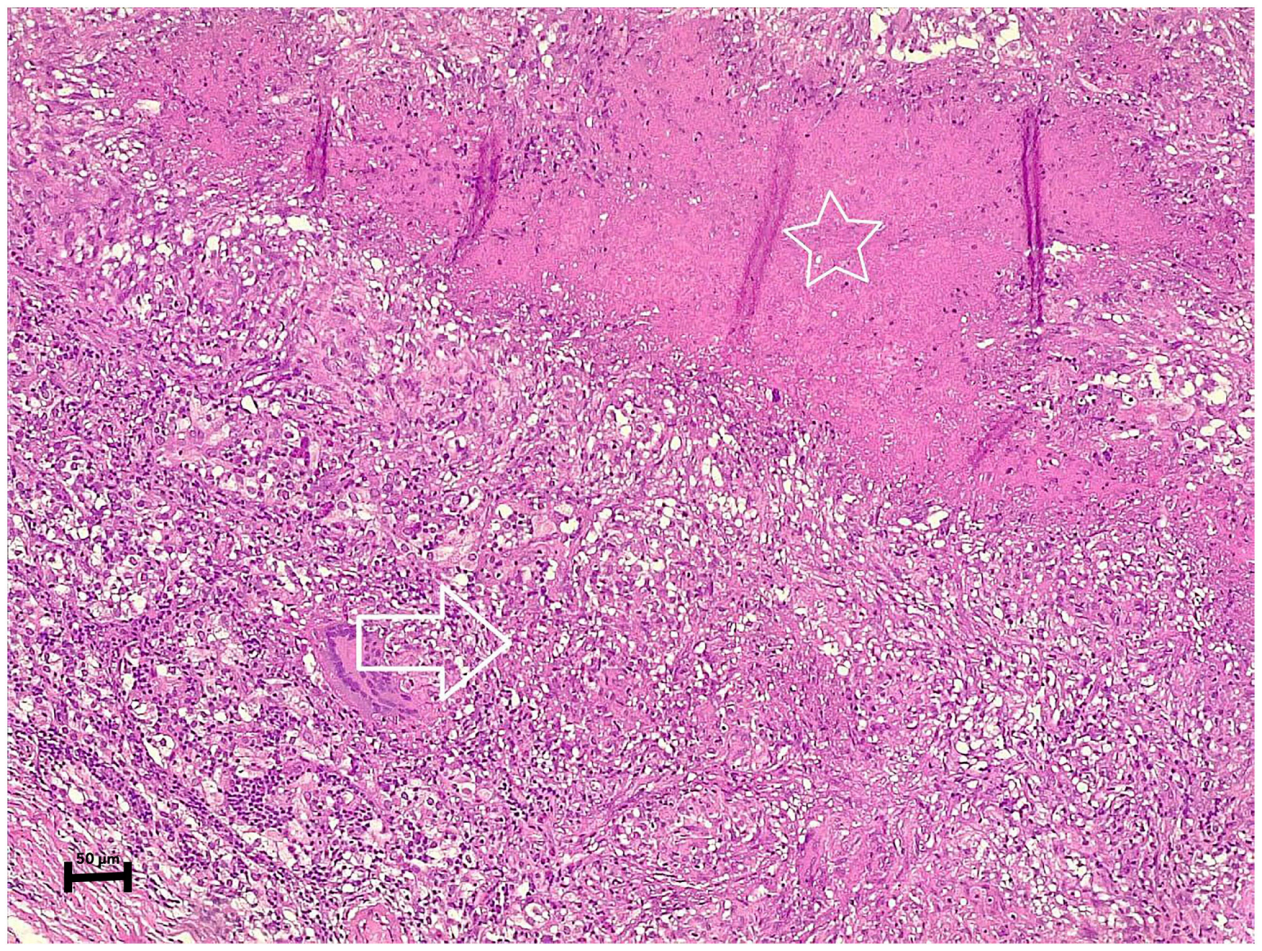
The section of the specimen (stained with eosin and hematoxylin) under microscopy illustrates a parathyroid adenoma containing well-defined epithelioid granulomas (arrow) with areas of caseating necrosis (star).

After proceeding to the left thyroid lobectomy and excising the left parathyroid gland, the histopathological examination revealed a parathyroid adenoma with caseating granulomatous inflammation involving the adenoma, suggestive of TB (Fig. 2). In addition, the left thyroid gland exhibited a nodular goiter with focal lymphocytic thyroiditis.

The histopathological examination was performed by the authors' laboratory as follows: The sections (4-µm-thick) were paraffin-embedded and fixed with 10% neutral buffered formalin at room temperature for 24 h. They were subsequently stained with hematoxylin and eosin (Bio Optica Co.) for 1-2 min at room temperature and then examined under a light microscope (Leica Microsystems GmbH).

### Follow-up

The post-operative period was uneventful, and the calcium level of the patient decreased to 10 mg/dl. A negative acid-fast bacillus test (AFB) of sputum, as well as negative findings from a chest X-ray were achieved post-operatively. Subsequently, the symptoms resolved following the initiation of treatment for TB (Rifampin, 600 mg, twice a day, for 6 months), and the generalized body aches subsided.

## Discussion

TB is a communicable disease that spreads by coughing and typically affects the lungs (pulmonary TB). Extrapulmonary TB may affect other parts of the body (15). The clinical symptoms of TB vary and rely on the host and microbe features along with the interaction between them. Extrapulmonary TB involvement tends to rise in immunocompromised patients (16). Hyperparathyroidism is categorized into three types, primary, secondary, and tertiary hyperparathyroidism. Parathyroid adenoma is the cause of almost 85% of primary hyperparathyroidism cases. Parathyroid adenoma is a type of parathyroid proliferative disease. Patients with primary hyperparathyroidism typically present with elevated blood calcium levels with high parathyroid hormone levels (17,18). However, the impact of primary TB on parathyroid function remains unknown. Granulomatous inflammation of endocrine organs usually leads to a decrease in the function of the affected gland (8). However, the case described herein presented with hyperparathyroidism along with granulomatous changes within the parathyroid adenoma, and the patient was immunocompetent.

Inflammatory conditions of the parathyroid gland and its hyperfunctioning are poorly defined subjects. Theories have suggested non-infectious and autoimmune disorders as an etiological factor for hyperparathyroidism (19). Compared to other endocrine organs, parathyroid gland inflammatory disorders are uncommon with an unknown pathophysiology (20,21). Granulomatous inflammation is a histological response of body tissue to cell damage from neoplastic conditions, infections, drugs, toxins, allergies, and autoimmune diseases (4). The case described in the present study was an incidental finding of parathyroid TB; the exact cause of hyperparathyroidism was uncertain, although the condition was suspected to be caused by infection as per the aforementioned theory.

Hypercalcemia is defined as a blood calcium level >10.50 mg/dl. Primary hyperparathyroidism, sarcoidosis and TB may induce hypercalcemia (17). In primary hyperparathyroidism, hypercalcemia occurs from osteoclast activation due to vitamin D-mediated granulomatous diseases (7). Parathyroid adenoma is a type of parathyroid proliferative disease in which patients usually have primary hyperparathyroidism and excessive blood calcium levels, resulting in weakness, polydipsia, polyuria and nephrolithiasis. Necrotizing granulomatous inflammation of the parathyroid with a functional adenoma is an unusual condition (4). The case described herein complained of generalized body aches and weakness, as well as recurrent renal calculi.

Acid-fast staining and mycobacteria culture remain the diagnostic tool for TB. Sputum is the most common specimen obtained for the diagnosis of pulmonary infection with TB (22). Good conventional chest radiography is still the major method for early diagnosis and the follow-up of patients with pulmonary TB. Despite normal radiographs, up to 10% of immunocompetent individuals have been observed to have TB (23). In the case presented herein, both the AFB test and conventional chest radiography postoperatively were negative. Primary pulmonary TB was thus ruled out.

The major limitations of the present study are as follows: First, an analysis of vitamin D levels was not performed for the patient. Second, there was a lack of a sestamibi parathyroid scan. Third, there was no post-operative bacterial culture for TB, vitamin D, and PTH hormone levels.

In conclusion, caseating granulomatous inflammation of the parathyroid gland caused by TB is an extremely rare condition. In TB-prevalent communities, TB may be considered when patients have symptoms of hypercalcemia. To the best of our knowledge, this is the eighth case described in the literature to date.

## Data Availability

The datasets used and/or analyzed during the current study are available from the corresponding author upon reasonable request.
